# Effects of power ultrasound on the activity and structure of β‐D‐glucosidase with potentially aroma‐enhancing capability

**DOI:** 10.1002/fsn3.1035

**Published:** 2019-05-15

**Authors:** Yujing Sun, Li Zeng, Yuanzhong Xue, Tianbao Yang, Zongming Cheng, Peilong Sun

**Affiliations:** ^1^ Department of Food Science and Technology Ocean College Zhejiang University of Technology Hangzhou China; ^2^ Food Quality Laboratory Agricultural Research Service, US Department of Agriculture Beltsville Agricultural Research Center Beltsville Maryland; ^3^ Department of Plant Sciences University of Tennessee Knoxville Tennessee

**Keywords:** β‐d‐Glucosidase, activity, enhancing aroma, structure, ultrasound

## Abstract

β‐d‐glucosidase can release aroma precursors to improve the flavor of plant food, but the hydrolysis efficiency of the enzyme is low; the purpose of this study was to improve the enzyme activity using ultrasound. The effects of ultrasound parameters on β‐d‐glucosidase activity were investigated, and the respective structures of enzyme activated and enzyme inhibited were further analyzed. Low temperature (20–45°C), low ultrasonic intensity (<181.53 W/cm^2^), and short treatment time (<15 min) led to the activation of β‐d‐glucosidase, whereas high temperature (45–60°C), high ultrasonic intensity (>181.53 W/cm^2^), and long treatment time (>15 min) led to its inhibition. Application of ultrasound lowered the optimum temperature for β‐d‐glucosidase activity from 50 to 40°C. Ultrasound did not change the primary structures of the enzyme, but changed the secondary structures. When ultrasound activated β‐d‐glucosidase, the α‐helix contents were increased, the β‐fold and irregular coil content were reduced. When ultrasound inhibited β‐d‐glucosidase, the contents of β‐folds were increased, the α‐helix and irregular coil contents were reduced.. In summary, activation or inhibition of β‐d‐glucosidase under ultrasound was determined by the ultrasound conditions. This study suggests that ultrasound combined with β‐D‐glucosidase can be used in aroma‐enhancing.

## 1 INTRODUCTION

Flavor compounds in fruits, occurring in their free forms, in which they contribute directly to fruit flavor, are also present in bound forms. Glycosidically bound aroma compounds are nonvolatile and odorless glycosides. Glycosides of volatile compounds identified in fruits are mainly *O*‐β‐d‐glucosides or *O*‐diglycosides, rarely trisaccharide glycoconjugates. Flavor glycoconjugates were firstly identified in roses (Francis & Allcock, 1969) and are present in many fruits, such as grapefruit (Dziadas & Jeleń Henryk, 2016; Hjelmeland & Ebeler, 2015), strawberry (Ubeda, Callejón, Troncoso, Morales, & Garcia‐Parrilla, 2014), citrus fruit (Igor, Josip, & Zvonimir, 2007; Ren et al., 2015), pineapple (Wu, Kuo, Hartman, Rosen, & Ho, 1991), peach (Krammer, Winterhalter, Schwab, & Schreier, 1991).

β‐d‐glucosidase is an important flavor‐related enzyme, and it can catalyze the hydrolysis of aryl and alkyl β‐D‐glucosides to remove β‐D‐glucose, thus enhancing the aroma profile of fruit products. They play a significant role in the release of many volatile compounds from their glycosidic precursors in fruits and vegetables. Hence, they have been applied in enhancing the flavor of fruit juices (Agrawal, Verma, & Satlewal, 2016; Tian et al., 2017) and fermented beverages (de Ovalle, Cavello, Brena, Cavalitto, & Gonzalez‐Pombo, 2018; Romo‐Sánchez, Arévalo‐Villena, Romero, Ramirez, & Pérez, 2014; Sharp, Steensels, & Shellhammer, 2017).

However, the method of β‐d‐glucosidase hydrolysis of glycosidically bound volatiles has a major drawback in that the hydrolysis time is too long which has limited its application in food industry. Thus, it is essential to improve the enzyme activity. Ultrasound is a potential method for modifying enzyme activity (Delgado‐Povedano & Castro, 2015; Huang, Chen, et al., 2017; Nadar & Rathod, 2017), and many researchers have used it to improve food enzymes. For example, Dalagnol, Silveira, Silva, Manfroi, and Rodrigues (2017) found that ultrasound improved the activities of pectinase, xylanase, and cellulase. Ma et al. (2011) found that energy‐gathered ultrasound improved the activity of alcalase by 5.8%. Barton, Bullock, and Weir (1996) found that ultrasound improved the activities of some glycosidase enzymes. Conversely, some researchers have used ultrasound to inhibit enzyme activity. Terefe et al. (2009) investigated the ultrasonic inactivation of polygalacturonase and pectin methylesterase in tomato juice at a frequency of 20 kHz and temperatures between 50 and 75°C. Rodrigues, Fernandes, García‐Pérez José, and Cárcel (2016) found that ultrasound‐assisted drying increased the activities of polyphenoloxidase, peroxidase, and peroxidase ascorbate in apples, but led to partial inactivation of pectin methylesterase. To the best of our knowledge, there have been no reports on the effects of ultrasound on β‐d‐glucosidase.

The objectives of this study were to evaluate the effects of ultrasonic power, time, temperature, medium pH, and duty cycle on β‐d‐glucosidase activity, to analyze the structures of the enzyme under its activation and inhibition conditions conducive to, and to explore the mechanism whereby ultrasound affects enzyme activity. The results could expedite the application of ultrasound in enhancing aroma in fruit juice.

## 2 MATERIALS AND METHODS

### 2.1 Chemicals

β‐d‐Glucosidase and *p*‐nitrophenol were purchased from Sigma‐Aldrich. *p*‐Nitrophenyl‐β‐d‐galactopyranoside (*p*NPG, 98%) was purchased from Aladdin Co. Anhydrous sodium carbonate, citric acid, trisodium citrate, monobasic sodium phosphate, and disodium hydrogen phosphate were purchased as analytical grade reagents from Shanghai Chemical Reagent Co.

### 2.2 Preparation of β‐d‐glucosidase solution

β‐d‐Glucosidase (5.0 mg) was added to citric acid–trisodium citrate buffer (40 ml; 0.1 M, pH 5) to prepare a 0.04 mmol enzyme solution, which was stored at −4°C prior to use.

### 2.3 β‐d‐glucosidase assay

β‐d‐Glucosidase activity was determined by UV/Vis spectrophotometry (UV‐2550; Shimadzu Corporation) as described by Barbagallo, Spagna, Palmeri, and Torriani (2004) with slight modifications. Enzyme solution (0.1 ml), pH 5.0 citric acid–trisodium citrate buffer (0.3 ml), and 5 mM *p*NPG (0.2 ml) were combined in a 10‐ml stopper tube, and the mixture was incubated for 10 min at 50°C. Thereafter, 1 M Na_2_CO_3_ solution (2 ml) was added to terminate the reaction, and the mixture was diluted to a fixed volume of 5 ml with distilled water. The absorbance of the yellow mixture was measured at 400 nm to determine the amount of *p*‐nitrophenol. β‐d‐Glucosidase activity was calculated according to the following formula:

(1) $$X=\frac{\mathrm{cV}}{tV^{\prime }}$$

where *X* is the β‐d‐glucosidase activity (U/ml), *c* is the concentration of *p*‐nitrophenol (mM), *V* is the final volume of reaction solution (ml), *V*′ is the volume of β‐d‐glucosidase solution (ml), and *t* is the reaction time (min).

### 2.4 Ultrasound treatment

Ultrasound treatment was carried out with a probe ultrasonic processor (Scientz‐IID; Ningbo Scientz Biotechnology Co.). Salient parameters of the probe ultrasonic processor were maximum power 950 W, frequency 25 kHz, diameter of horn microtip 2 mm. Aliquots (2 ml) of enzyme solution (prepared as described in Section 2.2) were added to glass tubes (1 cm diameter, 7 cm height), and then, these tubes were immersed in a low‐temperature water bath (DC‐1006; Safe Corporation) at a preset constant temperature and subjected to ultrasound treatment.

The main effects of different factors of ultrasound treatment on the activity of β‐d‐glucosidase were tested by single‐factor experiment design. The variables examined with regard to their effect on enzyme activity were duty cycle (33.33, 40, 50, 66.67, and 100%), temperature (20, 30, 40, 50, and 60°C), time (1, 5, 10, 15, 20, and 25 min), electrical acoustic intensity (ranging from 0 to 484.08 W/cm^2^), and pH (3, 4, 5, 6, and 7).

Apart from the specific ultrasound conditions, as mentioned in the Results Section, the general ultrasound conditions were kept constant. The probe was placed 0.5 cm from the top surface of the extraction cell. The liquid height, measured as the distance from the horn microtip to the bottom of the tube, was 1.5 cm. The temperature was 40°C, pulsed mode was applied (1s on and 2s off), the treatment time was 10 min, the pH was 5, and the ultrasonic intensity was 60.51 W/cm^2^.

### 2.5 Calculation of electrical acoustic intensity

The electrical acoustic intensity dissipated from the probe microtip was calculated according to the following formula (Li, Pordesimo, & Weiss, 2004):

(2) $$I=\frac{P}{\pi r^{2}}$$

where *r* is the radius of the probe microtip and *P* is the input power. In the present study, the input power levels were adjusted to 1%, 3%, 5%, 10%, 20%, 30%, and 40% of the maximum input power (950 W), equating to 9.5, 28.5, 47.5, 95.0, 190, 285, and 380.0 W, respectively. The corresponding ultrasonic intensities were 12.10, 36.31, 60.51, 121.02, 242.04, 363.06, and 484.08 W/cm^2^, respectively.

### 2.6 Structural characteristics of β‐D‐glucosidase treated by ultrasound

#### 2.6.1 Circular dichroism (CD)

β‐d‐Glucosidase solution was sonicated, and its circular dichroism spectrum was measured with respect to buffer solution as the blank in a quartz cuvette of 1 mm optical path length at room temperature (20 ± 1°C). Scanning was conducted in the far‐UV range of 185–290 nm at 200 nm/min, with 1 nm as spectral spacing (Subhedar & Gogate, 2014). CD data are expressed in the form of mean residue ellipticity [θ] deg cm^2^/dmol. The secondary structures (α‐helices, β‐turns, and β‐sheets) of β‐d‐glucosidase, with or without ultrasound treatment, were analyzed using Dichroweb (Ma et al., 2015).

#### 2.6.2 Fluorescence spectroscopy

Intrinsic fluorescence spectra of β‐d‐glucosidase solution, untreated or treated with ultrasound, were recorded at room temperature (20 ± 1°C) on a fluorescence spectrophotometer (Varian Inc.; Model Cary Eclipse) at 280 nm (excitation wavelength, slit 5 nm) and 290–500 nm (emission wavelength, slit 5 nm), at a scanning speed of 1000 nm/s. The buffer used to dissolve β‐d‐glucosidase served as the blank (Huang, Wei, Mo, Wang, & Ma, 2017).

#### 2.6.3 UV/Vis spectrophotometry

β‐d‐Glucosidase solution was sonicated. The UV/Vis absorption spectrum was measured on a UV/Vis spectrophotometer (UV‐2550; Shimadzu Corporation), scanning over the wavelength range 800–200 nm, sampling at intervals of 1.0 nm and room temperature (20 ± 1°C) (Pellicer & Gómez‐López, 2017).

### 2.7 Statistical analyses

Experiments were performed at least twice, and measurements were made in triplicate. Data are expressed as mean ± standard deviation (*SD*). Regression analyses and other statistical analyses were performed using Microsoft Excel 2016 and the SPSS program, version 17.0. Means were compared by Duncan's new multiple range test. Statistically significant differences were set at *p *<* *0.05.

## 3 RESULTS AND DISCUSSION

### 3.1 Effect of ultrasonic intensity on the activity of β‐d‐glucosidase

**Figure 1:**
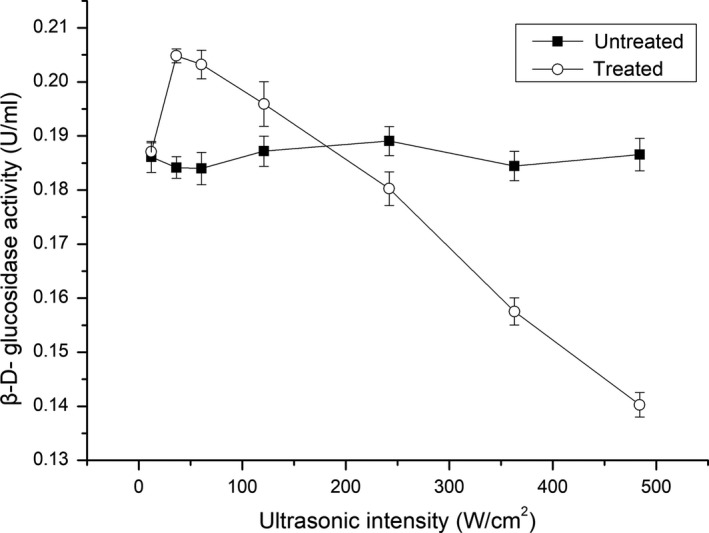
Effect of ultrasonic intensity on the activity of β‐D‐glucosidase

Figure 1 shows the effect of ultrasonic intensity on β‐d‐glucosidase activity. The β‐d‐glucosidase activity was improved by ultrasonic intensity in the range from 0 to 181.53 W/cm^2^ (*p *<* *0.05) compared with the untreated enzyme. However, its activity was inhibited by ultrasonic intensity in the range from 181.53 to 484.08 W/cm^2^ (*p *<* *0.05). The highest enzyme activity was achieved at 12.10 W/cm^2^.

The results indicated that ultrasonic intensity is an important factor with regard to enhancing or inhibitory effects of ultrasound on enzyme activity. Under the condition of low intensity, ultrasound generates cavitation, magnetostrictive effects, and mechanical oscillation, which affect the conformation of the enzyme and increase its contact with the substrate. Conversely, high‐intensity ultrasound inhibited the enzyme activity, which may be due to the reaction of generated hydroxyl or hydrogen radicals with the protein backbone. This could in turn lead to enzyme aggregation, thereby obstructing the active sites.

### 3.2 Effect of ultrasonication time on the activity of β‐d‐glucosidase

**Figure 2:**
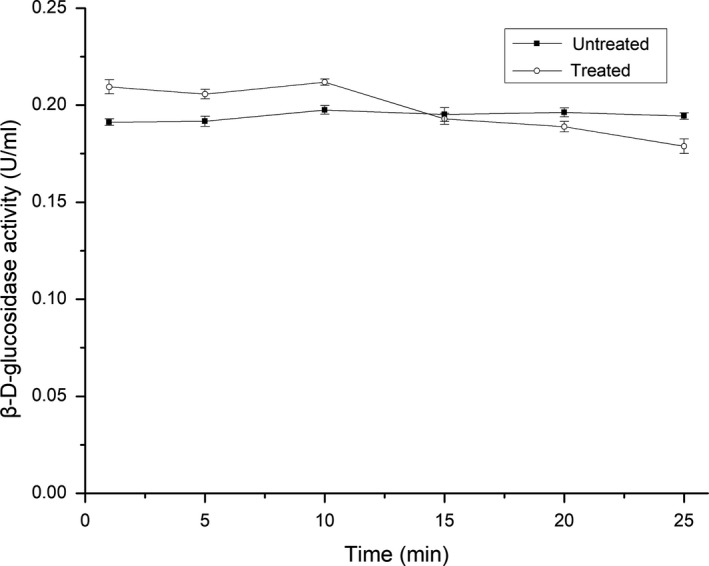
Effect of ultrasonication time on the activity of β‐D‐glucosidase

The effect of ultrasonication time on the activity of β‐d‐glucosidase is shown in Figure 2. The β‐d‐glucosidase activity was improved compared with the untreated enzyme at treatment time ranging from 0 to 15 min (*p *<* *0.05), but was inhibited at treatment times ranging from 15 to 25 min (*p *<* *0.05). The results may be explained that short‐term ultrasound treatment at low power intensity changes the conformation and causes more active sites to appear on the surface of the enzyme, whereas long‐term ultrasound treatment, even at the appropriate power intensity, can still destroy the conformation.

### 3.3 Effect of ultrasonication temperature on the activity of β‐d‐glucosidase

**Figure 3:**
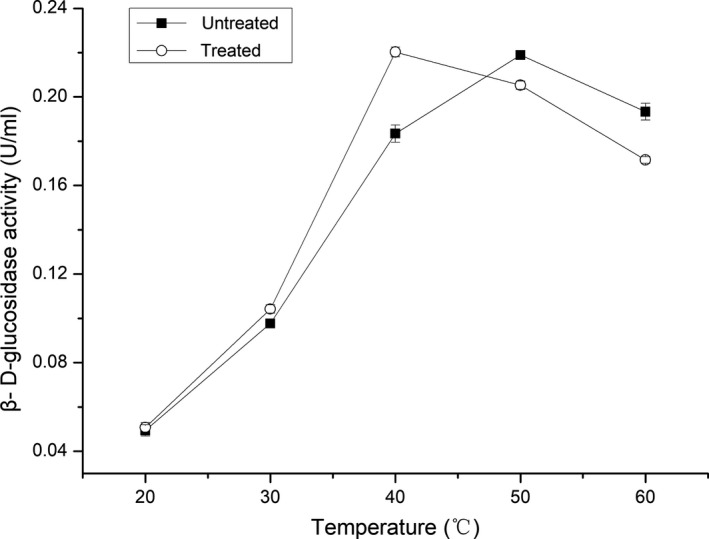
Effect of temperature on the activity of β‐D‐glucosidase under ultrasound

The optimum temperature for β‐d‐glucosidase activity under untreated conditions was found to be 50°C, but this was lowered to 40°C following ultrasonic treatment (Figure 3). Compared to the untreated enzyme, β‐d‐glucosidase activity was improved at temperatures between 20 and 45°C, but inhibited at higher temperatures. These results indicated that ultrasound treatment could stimulate β‐d‐glucosidase activity at low temperature, but impair it at high temperature, thus changing the optimum temperature. This finding is in contrast to previous literature reports. For example, Wang, Cao, Sun, Wang, and Mo (2011) found that ultrasound improved alliinase activity within the tested temperature range and did not affect the optimal temperature for enzyme activity. Liu et al. (2008) reported that ultrasonic treatment raised the optimal temperature for lipase activity by about 5–10°C. These differences may be attributed to the different characteristics of the enzymes.

### 3.4 Effect of pH on the activity of β‐d‐glucosidase under ultrasonication

**Figure 4:**
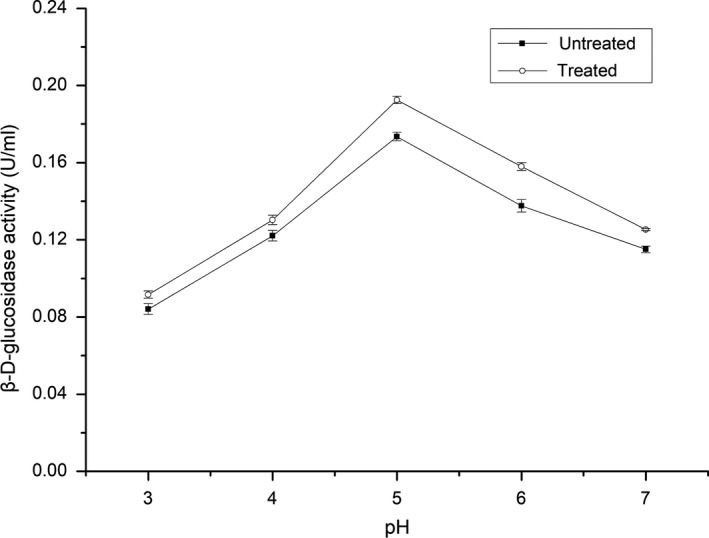
Effect of pH on the activity of β‐D‐glucosidase under ultrasound

pH is another key factor affecting enzyme activity. Higher or lower pH can lead to partial or complete inactivation of an enzyme. The effect of ultrasound on optimal pH is plotted in Figure 4. For the enzyme under ultrasound irradiation, pH 5 was identified as optimal, consistent with that without ultrasound treatment. This result suggested that ultrasound did not change the optimum pH of β‐d‐glucosidase and improved its activity under the studied pH conditions. It is well known that pH can influence the ionization state of an enzyme, modifying its active site. The optimal pH conditions in the presence and absence of ultrasound in this study were similar, implying that ultrasound caused little change to the ionization state of β‐d‐glucosidase.

### 3.5 Effect of duty cycle on the activity of β‐d‐glucosidase

**Figure 5:**
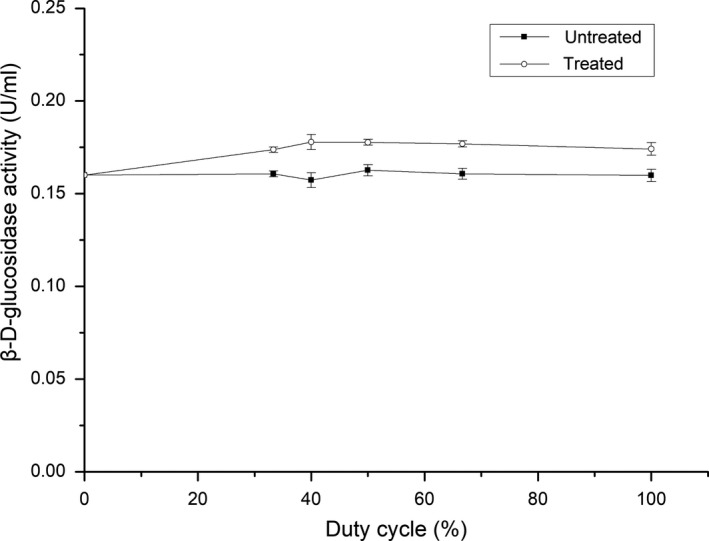
Effect of duty cycle on the activity of β‐D‐glucosidase

Figure 5 shows the effect of ultrasound duty cycle on β‐d‐glucosidase activity. It can be seen that β‐d‐glucosidase activity was improved when the duty cycle ranged from 33.33% to 100% compared to the untreated sample. Specifically, it first increased when the duty cycle ranged from 33.33% to 40%, then showed a slight decrease as the duty cycle ranged from 40% to 100%. This can be explained that the extensive cavitation at higher duty cycle affected the structure of the protein. It is well known that continuous ultrasound has a damaging effect on ultrasound transducers, because the heat caused by cavitation cannot be completely dissipated in a short time. Therefore, to protect equipment and save energy, a low duty cycle of 40% would seem to be a good choice for the activation of β‐d‐glucosidase.

### 3.6 Structural characteristics of β‐d‐glucosidase treated by ultrasound

#### 3.6.1 Circular dichroism(CD) analysis

**Figure 6:**
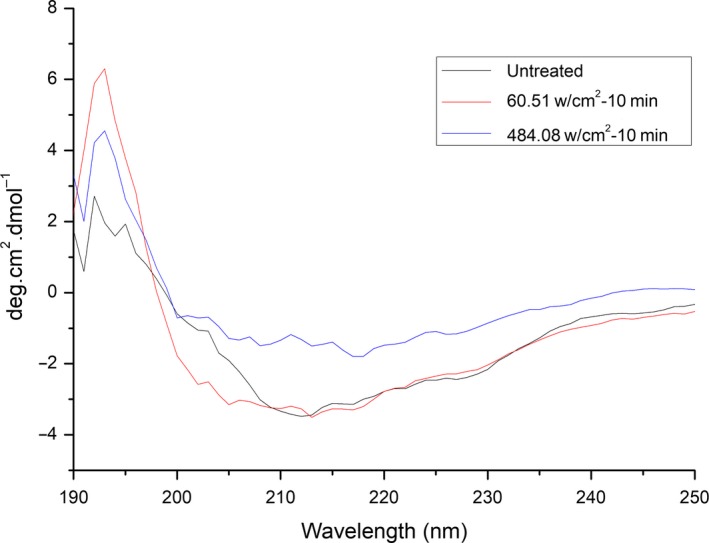
Secondary structures of β‐D‐glucosidase treated by ultrasound

CD spectroscopy is the most widely applied technique for obtaining information about enzyme secondary structure. The contents of α‐helices, β‐sheets, β‐turns, and random coils in β‐d‐glucosidase were determined to describe the relationship between the enzyme activity and its secondary structure. Figure 6 shows the CD spectra of control and ultrasound‐treated β‐d‐glucosidase at 60.51 W/cm^2^ (activation) and 484.08 W/cm^2^ (inhibition). The spectrum of the control displayed a positive band at 192 nm and two negative bands at 212 and 221 nm, characteristic of an α‐helical structure (Liu, Yan, Cao, Chong, & Lü, 2014). The spectrum also featured a positive band at 190 nm and a negative band at 217 nm, characteristic of a β‐sheet structure (Pouderoyen, Snijder, Benen, & Dijkstra, 2003). Further, weak positive bands at 205 nm and 220–240 nm are characteristic of β‐turn and random coil structures, respectively (Pouderoyen et al., 2003). After treated with 60.51 W/cm^2^ ultrasound, the positive band at 190 nm almost disappeared and the negative band at 217 nm decreased, indicating that 60.51 W/cm^2^ ultrasound decreased the β‐sheet content of the enzyme. After treatment with 484.08 W/cm^2^ ultrasound, the two negative bands at 212 and 221 nm had decreased, indicating that 484.08 W/cm^2^ ultrasound decreased the α‐helix content. Moreover, the positive band at 190 nm and the negative band at 217 nm had increased, indicating that 484.08 W/cm^2^ ultrasound increased the β‐sheet content of the enzyme. The positive bands at 205 nm and 220–240 nm were not significantly changed by the respective ultrasound treatments, suggesting that ultrasound did not affect the β‐turn and random coil contents of the enzyme.

The contents of α‐helices, β‐sheets, and random coils of β‐d‐glucosidase were calculated by means of Dichroweb (Ma et al., 2015) (Table 1). Sonication of β‐d‐glucosidase at 60.51 W/cm^2^ increased the α‐helix content by 50% and decreased the β‐sheet content by 18%. Sonication of β‐d‐glucosidase at 484.08 W/cm^2^ decreased the α‐helix content by 40% and increased the β‐sheet content by 39%. The results suggest that the active site of β‐d‐glucosidase may lie within an α‐helix. The changes of enzyme secondary structure under ultrasound may be due to the high temperature and high pressure of cavitation, and the mechanism needs future studies.

**Table 1:** Effect of ultrasound on β‐d‐glucosidase secondary structure

Sample	α‐Helical/%	β‐Sheet/%	β‐Turn/%	Random coil/%
Control	22.8	25.5	22.2	29.1
Sonicated (low intensity, 60.51 w/cm^2^)	31.7	20.9	22.6	26
Sonicated (high intensity, 484.08 w/cm^2^)	12.6	35.6	23.3	27.5

#### 3.6.2 Fluorescence spectrometric analysis

**Figure 7:**
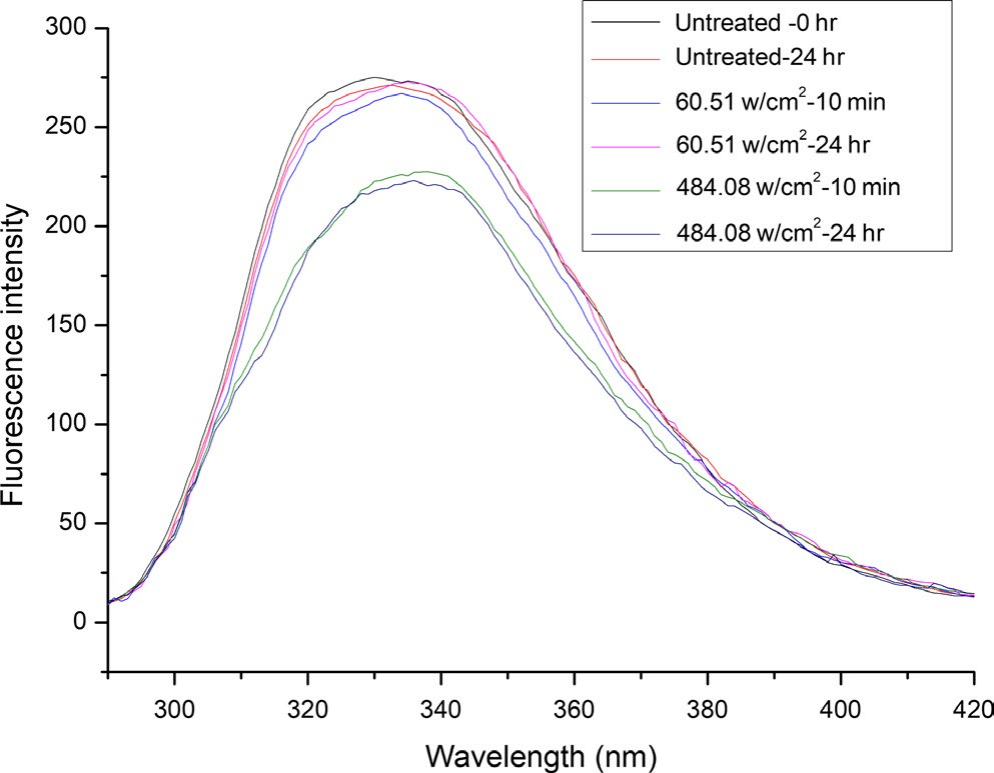
Fluorescence spectra of β‐D‐glucosidase treated by ultrasound

Fluorescence spectroscopy is a useful technique for following tertiary structural transitions in proteins, because the intrinsic fluorescence of aromatic amino acid residues (such as Trp, Tyr, and Phe residues, particularly Trp) is sensitive to the polarity of microenvironments along the transition (Zhao & Yang, 2008). As shown in Figure 7, the emission fluorescence spectra of untreated and ultrasonically treated β‐d‐glucosidase each featured a peak at 330 nm. After treatment with 484.08 W/cm^2^ ultrasound, the fluorescence intensity decreased significantly (from 275.0 to 227.5) and the peak was red‐shifted (from 330 to 338 nm). After treatment with 60.51 W/cm^2^ ultrasound, the intensity showed a weak decrease (275.0–267.0) and the peak was slightly red‐shifted (from 330 to 334 nm). The fluorescence intensity recovered to the original level of the native enzyme when the sample treated with 60.51 W/cm^2^ ultrasound was kept for 24 hr at 4°C. However, the fluorescence intensity did not change with time when the sample treated with 484.08 W/cm^2^ ultrasound was kept for 24 hr at 4°C. The results suggest that β‐d‐glucosidase has a flexible structure. The effect of low‐intensity ultrasound on the enzyme is reversible, but the effect of high‐intensity ultrasound is irreversible.

#### 3.6.3 UV/Vis spectroscopic analysis

**Figure 8:**
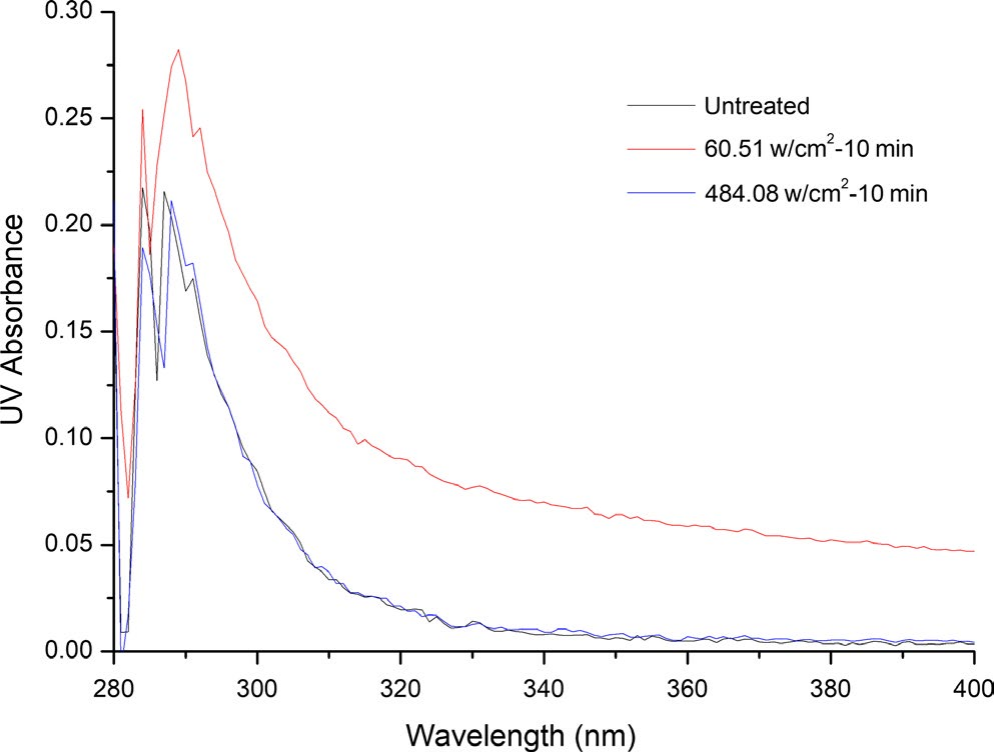
UV absorption spectroscopy of β‐D‐glucosidase treated by ultrasound

Figure 8 shows the UV absorption spectra of β‐d‐glucosidase before and after treatment with ultrasound. Absorption peaks of β‐d‐glucosidase are seen at 284, 287, and 291 nm. The UV/Vis absorption spectrum of β‐d‐glucosidase did not change after treatment with ultrasound at 60.51 or 484.08 W/cm^2^. The results indicated that ultrasound did not change the primary molecular structure, molecular composition, or chemical bonding of β‐d‐glucosidase. However, the UV absorption intensity of β‐d‐glucosidase treated with 484.08 W/cm^2^ ultrasound was significantly increased compared to the control and the sample treated with 60.51 W/cm^2^ ultrasound. This can be explained that high‐intensity ultrasound increased the content of soluble protein and made more chromophores appear on the surface of the enzyme (Zhang, Claver, Zhu, & Zhou, 2011).

## 4 CONCLUSIONS

In summary, the effects of ultrasound on β‐d‐glucosidase activity were studied, and the mechanism whereby ultrasound affects enzyme activity was further explored. It was found that temperature, ultrasonic intensity, and treatment time determined the activation or inhibition of β‐d‐glucosidase, whereas duty cycle and pH only impacted the effects. The activation or inhibition of β‐d‐glucosidase under ultrasound may be attributed to changes in secondary structure. For activation, ultrasound increased the content of α‐helices, decreased the content of β‐folds and irregular coils of β‐d‐glucosidase. For inhibition, ultrasound increased the content of β‐folds and reduced the content of α‐helices and irregular coils. This study gives very useful information concerning the application of the ultrasound technique for enhancing aroma in fruit juice.

## CONFLICT OF INTEREST

The authors declare that they do not have any conflict of interest.

## ETHICAL REVIEW

This study does not involve any human or animal testing.

## INFORMED CONSENT

Written informed consent was obtained from all study participants.
